# Safety Profile of Antipsychotic Drugs: Analysis Based on a Provincial Spontaneous Reporting Systems Database

**DOI:** 10.3389/fphar.2022.848472

**Published:** 2022-03-09

**Authors:** Kangyuan Guo, Zhanchun Feng, Shanquan Chen, Ziqi Yan, Zhiming Jiao, Da Feng

**Affiliations:** ^1^ School of Medicine and Health Management, Tongji Medical College, Huazhong University of Science and Technology, Wuhan, China; ^2^ Department of Psychiatry, University of Cambridge, Cambridge, United Kingdom; ^3^ School of Pharmacy, Tongji Medical College, Huazhong University of Science and Technology, Wuhan, China

**Keywords:** antipsychotics, adverse drug reaction, spontaneous reporting system, signal mining, risk factor, pharmacovigilance, schizophrenia

## Abstract

**Introduction:** Antipsychotic drugs are the main therapy for schizophrenia and have been widely used in mental disorder fields. However, the research on the safety of antipsychotic drugs in the real-world is rare. The purpose of this research is to evaluate the safety of antipsychotic drugs based on real-world data.

**Methods:** ADR reports collected by the Henan Adverse Drug Reaction Monitoring Center from 2016 to 2020 were analyzed. We described the safety of antipsychotic drugs by descriptive analysis and four signal mining methods. Meanwhile, the risk factors for serious adverse reactions of antipsychotics were identified.

**Results:** A total of 3363 ADR reports related to antipsychotics were included. We found that the number of adverse drug reaction reports and the proportion of serious adverse reactions have increased year by year from 2016 to 2020. Most adverse drug reactions occurred within 3 months after taking the medicine. The symptoms caused by typical antipsychotics and atypical antipsychotics were different and dyskinesia was more common in typical antipsychotics. Most patients improved or recovered after treatment or intervention while only one patient had sequelae. Low-level hospitals, psychiatric hospitals, youth, and old age could increase the risk of serious adverse reactions. Four off-label signals were found through signal mining, including amisulpride-pollakiuria, ziprasidone-dyspnoea, quetiapine-urinary incontinence, olanzapine-hepatic function abnormal.

**Conclusion:** We found that most ADRs occurred within 3 months after taking the medicine, so close observation was required for patients during the first 3 months of treatment. The ADRs of antipsychotics involved multiple organ-system damages but were not serious. It might be recommended to take alternative drugs after a serious ADR occurred. The symptoms caused by typical APDs and atypical APDs were different. For patients with typical APDs, dyskinesia was more common and should be given special attention. Statistics showed that low-level hospitals, psychiatric hospitals, youth, and old age were risk factors for serious ADRs. The four off-label signals obtained by signal mining should be paid special attention, including amisulpride-pollakiuria, ziprasidone-dyspnoea, quetiapine-urinary incontinence, and olanzapine-hepatic function abnormal.

## Introduction

Schizophrenia is a severe emotional disorder marked by delusions and hallucinations, cognitive impairment, and blunted affect, which threatens health and causes a heavy economic burden for the patients. The Global Burden of disease Study 2016 showed that the economic burden of schizophrenia ranked 12th among 310 diseases and injuries in the world (Vos et al., 2017). In China, the lifetime prevalence of schizophrenia is 6‰ with approximately 8.4 million (Huang et al., 2019). Antipsychotic drugs (APDs) are the main therapy for schizophrenia, which maintain the stability of disease remission, improves patients’ social function, attain the purpose of recovery and return to society (Gaebel et al., 2011; Sampson et al., 2013; De Hert et al., 2015). However, long-term APDs treatment is associated with many adverse reactions, such as weight gain, sexual dysfunction, akathisia, extrapyramidal disorder, orthostatic hypotension, hyperprolactinemia, etc (Muench and Hamer, 2010; Young et al., 2015; Ames et al., 2016). According to World Health Organization (WHO), adverse drug reactions (ADRs) refer to a response to a drug that is noxious and unintended, and which occurs at doses normally used in man for the prophylaxis, diagnosis, or therapy of disease, or for the modification of physiological function (Edwards and Aronson, 2000). It has been reported the ADRs of APDs will negatively affect the patient s’ medication compliance, aggravate the patient s’ condition, and even increase the risk of certain diseases (Manu et al., 2015; Mouton et al., 2016). Hert reported that weight gain, cardiovascular and metabolic abnormalities caused by APDs might increase the risk of obesity, diabetes, and related cardiovascular diseases in schizophrenia patients (De Hert et al., 2012).

Currently, studies on the safety of APDs mainly focused on clinical trials. But the research based on real-world ADRs data is rare. Clinical trials have strict eligibility criteria, which may prohibit the inclusion of patients with multiple morbidities, and certain patient profiles may not have been represented, which might further limit the generalizability of these trials (Harpaz et al., 2012). Researches based on real-world data could sufficiently appreciate the risks and benefits of the medication, which might improve treatment decisions made by patients and their providers and support regulatory decision-making (Franklin et al., 2019). In this context, a safety evaluation of antipsychotics based on real-world data is warranted. Previous researches based on the real-world spontaneous reporting system database has focused on certain side effects or a few kinds of antipsychotics. Kato evaluated the relationship between antipsychotic drugs and adverse hyperglycemic events by using the FDA Adverse Event Reporting System database (Kato et al., 2015). McLean found that quetiapine treatment was related to alopecia based on the analysis of case reports from the New Zealand Intensive Medicines Monitoring Programme (McLean and Harrison-Woolrych, 2007). Sakai conducted a disproportionality analysis of second-generation antipsychotic exposure during pregnancy using the Japanese Adverse Drug Event Report database and found a potential signal for miscarriage for aripiprazole (Sakai et al., 2017). Although some studies have been carried out on antipsychotics based on the spontaneous reporting system database, the overall safety assessment of antipsychotics is still lacking. We performed a overall safety evaluation of APDs based on the spontaneous reporting system (SRS) of Henan Province in China. The purpose of this research was to study the safety of APDs from different perspectives of ADRs.

## Materials and Methods

### Data Source and Preprocessing

ADR reports come from the SRS in Henan Province, China, which includes basic information of patients, drug usage, symptoms, severity, and outcome of ADRs. In the reports, suspected drugs refer to drugs related to the occurrence of ADRs. In addition to suspected drugs, other drugs used by patients are concomitant drugs. The relationship between drugs and ADR was evaluated as certain, probable, possible, unlikely, or impossible.

The inclusion criteria are as follows:1) Reports were reported and entered into the system between 2016 and 2020; 2) Reports in which APDs were considered as suspected drugs; 3) Reports in which the relationship between APDs and ADRs was evaluated as certain, probable, or possible.

The exclusion criteria are as follows: 1) Reports were reported and entered into the system before 2016 or after 2020; 2) Reports didn’t involve APDs or in which APDs were considered as concomitant drugs; 3) Reports in which the relationship between APDs and ADRs was evaluated as unlikely or impossible.

The data were cleaned and preprocessed to ensure that they were clean and complete. In this research, APDs were defined as any drug of the N05A Anatomical-Therapeutic-Chemical (ATC) code group, which is a drug classification system developed by World Health Organization Collaborating Centre (WHO Collaborating Centre for Drug Statistics Methodology, 2021). Since there was no unified standard for drug names and ADR names in the reports, the drug names in the ATC classification system were used as the standard to unify the generic names, and the ADRs and clinical manifestations were unified based on Medical Dictionary for Regulatory Activities (MedDRA). According to The Administrative Measures on Reporting and Monitoring of ADRs, ADRs were divided into serious and non-serious ADRs. Serious ADRs result in death, life-threatening effects, cancer, a congenital anomaly, birth defects, significant or permanent human disability, damage to organ function, hospitalization or prolonged hospitalization or events that require intervention and treatment to avoid the above results (China, N.H.C.o.t.P.s.R.o., 2021). The hospital levels were classified into three levels according to the Measures for the Administration of the Hospital Grade. The level 1 hospital are primary healthcare institutions that directly provide comprehensive services of medical treatment, prevention, rehabilitation, and healthcare to the community. The level 2 hospital are regional hospitals that provide medical and health services across several communities and are technical centers for regional medical prevention. The level 3 hospital is a medical prevention technology center with comprehensive medical, teaching, and scientific research capabilities providing across provinces and cities medical and health services to the whole country (China, N.H.C.o.t.P.s.R.o., 2016). Antipsychotic drugs were divided into two types, typical and atypical (Meltzer, 2013). The definition for polypharmacy included the use of three or more medications. Since there may be more than one ADR in a report, each report was divided into multiple drug-ADR combinations before signal mining.

### Data Analysis

A descriptive analysis of gender, age, reporting year, drug, severity, and outcome of ADRs in the reports was performed. Logistic regression analysis was used to calculate the adjusted odds ratio. All data analyses were performed using SPSS 24.0 (IBM Corp. Armonk, NY). The level of significance was set at *p* < 0.05 (two-tailed).

In this paper, We used four signal mining methods for generating potential safety signals, including reporting odds ratio (ROR) (van Puijenbroek et al., 1999; van Puijenbroek et al., 2000), proportional reporting ratio (PRR) (van Puijenbroek et al., 2002), the method employed by the United Kingdom Medicines and Healthcare products Regulatory Agency using the PRR and chi-squared (abbreviated as ‘MHRA’ in this study) (Evans et al., 2001), and Bayesian Confidence Propagation Neural Network (BCPNN) (Bate et al., 1998; Orre et al., 2000). Table 1 shows how to calculate the counts of each drug-ADR combination. Then, signal mining was performed according to the formulas and criteria in Table 2.

**TABLE 1:** The fourfold table used in data mining.

Category of drugs	Target ADR N	Other ADRs N	Sum
Target drug	a	b	a+b
Other drugs	c	d	c + d
Sum	a+c	b + d	n = a+b + c + d

**TABLE 2:** Formulas and criteria for signal mining.

Method	Formula	Criteria
ROR	$ROR=\frac{\text{a}/\text{c }}{b/d}\;$	a≥3 and lower limit 95%CI > 1
	$SE\left(lnROR\right)=\sqrt{\left(\frac{1}{a}+\frac{1}{b}+\frac{1}{c}+\frac{1}{d}\right)}$	
	$95\%CI=e^{lnROR\pm 1.96SE\left(lnROR\right)}$	
PRR	$PRR=\frac{a/\left(a+b\right)}{c/\left(c+d\right)}$ $SE\left(lnPRR\right)=\sqrt{\left(\frac{1}{a}-\frac{1}{a+b}+\frac{1}{c}-\frac{1}{c+d}\right)}$ $95\%CI=e^{lnPRR\pm 1.96SE\left(lnPRR\right)}$	a≥3 and lower limit 95%CI > 1
MHRA	$PRR=\frac{a/\left(a+b\right)}{c/\left(c+d\right)}$	a≥3,PRR≥2 and χ2≥4
	$\chi^{2}=\frac{{\left(\left|ad-bc\right|-n/2\right)}^{2}n}{\left(a+b\right)\left(c+d\right)\left(a+c\right)\left(b+d\right)}\;\;$	
	OR $\chi^{2}=\frac{{\left(ad-bc\right)}^{2}n}{\left(a+b\right)\left(c+d\right)\left(a+c\right)\left(b+d\right)}$	
BCPNN	$E\left(IC\right)=\log_{2}\left(\frac{\left(a+1\right){\left(n+2\right)}^{2}}{\left(n+4\right)\left(a+b+1\right)\left(a+c+1\right)}\right))$	lower limit 95%CI > 0
	$V\left(IC\right)={\left(\ln2\right)}^{-2}\left(\frac{n-a+3}{\left(a+1\right)\left(n+5\right)}+\frac{n-\left(a+b\right)+1}{\left(a+b+1\right)\left(n+3\right)}+\frac{n-\left(a+c\right)+1}{\left(a+c+1\right)\left(n+3\right)}\right)$ $95\%CI=E\left(IC\right)\pm 2\sqrt{V\left(IC\right)}$	

## Results

### Basic Information of the Reports

**FIGURE 1:**
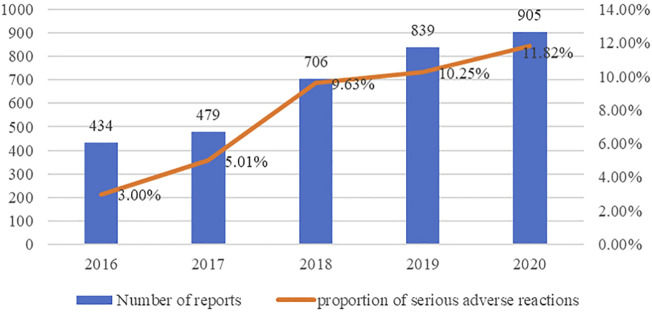
number of reports and proportion of serious ADRs in each year.

According to the inclusion and exclusion criteria, 3,363 reports were finally included from 570,000 ADR reports, with a male/female ratio of 1.10 and an average age of 33.29 ± 16.651 years. Most patients suffered from schizophrenia. In particular, we found that the number of reports and proportion of serious ADRs increased year by year from 2016 to 2020. This trend can be found in Figure 1.

### Characteristics of ADR

**FIGURE 2:**
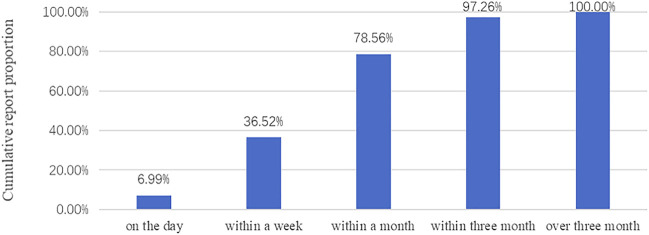
Occurrence time of ADRs.

Figure 2 shows the occurrence time of ADRs after starting the medication. Most ADRs occurred within 3 months after taking the medicine. Only a small amount of ADR occurred on the day.

3,363 reports related to 15 antipsychotic drugs, which were divided into two categories: typical and atypical. There were seven typical and eight atypical antipsychotics. Atypical APDs reported more reports of ADRs (2,970) and a higher proportion of severe ADRs (9.26%). In particular, we found that risperidone has the largest number of reports and chlorpromazine has the highest proportion of severe ADRs. See Table 3 for details.

**TABLE 3:** Drugs of the reports.

Drug type	Drug	Non-serious	Serious	Sum
atypical		2,695 (90.74%)	275 (9.26%)	2,970
	risperidone	984 (90.86%)	99 (9.14%)	1,083
	clozapine	497 (89.71%)	57 (10.29%)	554
	olanzapine	321 (91.71%)	29 (8.29%)	350
	Aripiprazole	220 (93.22%)	16 (6.78%)	236
	quetiapine	189 (88.32%)	25 (11.68%)	214
	amisulpride	191 (92.27%)	16 (7.73%)	207
	ziprasidone	180 (87.38%)	26 (12.62%)	206
	perospirone	113 (94.17%)	7 (5.83%)	120
typical		370 (94.15%)	23 (5.85%)	393
	haloperidol	211 (97.69%)	5 (2.31%)	216
	perphenazine	57 (89.06%)	7 (10.94%)	64
	sulpiride	57 (93.44%)	4 (6.56%)	61
	chlorpromazine	36 (83.72%)	7 (16.28%)	43
	penfluridol	5 (100.00%)		5
	chlorprothixene	3 (100.00%)		3
	droperidol	1 (100.00%)		1

Since there may be more than one ADR in a report, 3,363 reports were divided into 3,953 drug-ADR combinations. The symptoms and system-organ damages related to ADRs of antipsychotics were counted. The symptoms were unified based on Preferred Term (PT) in MedDRA and system-organ damages were unified based on primary system organ class (primary SOC). Statistics showed that 3,953 drug-ADR combinations involved a total of 20 system-organ damage, which mainly includes nervous system disorders (47.43%), gastrointestinal disorders (11.99%), and cardiac disorders (7.24%). Investigations (11.38%) indicate that various inspections are abnormal, but there is no clear system organ damage. Table 4 listed top 10 primary SOC.

**TABLE 4:** Number and percentage of ADRs related to primary SOC(TOP10).

SOC	Frequency	Percentage (%)
Nervous system disorders	1875	47.43
Gastrointestinal disorders	474	11.99
Investigations	450	11.38
Cardiac disorders	286	7.24
Psychiatric disorders	149	3.77
General disorders and administration site conditions	108	2.73
Skin and subcutaneous tissue disorders	97	2.45
Hepatobiliary disorders	94	2.38
Eye disorders	92	2.33
Metabolism and nutrition disorders	87	2.20

A total of 238 ADRs were identified. Extrapyramidal disorder (23.96%) was the most common symptom, followed by akathisia (5.94%) and constipation (5.01%). The ADR symptoms of typical and atypical APDs were different. Dystonia, abnormal sensation in eye, hypertonia, dry mouth, orthostatic hypotension, and insomnia were common ADR symptoms in typical APDs. Constipation, white blood cell count decreased, hepatic function abnormal, dizziness, and tachycardia were more common in atypical APDs. Table 5 listed top 10 symptoms. In this paper, we differentiated akathisia from extrapyramidal disorder based on the original description of the reports. Patients described as extrapyramidal disorder by the reporting agency experienced multiple extrapyramidal symptoms at the same time, such as dystonia, restlessness, and akathisia. Patients described as akathisia usually experienced only one symptom. Based on the above differences, we separate statistics for the two adverse reactions.

**TABLE 5:** Frequently reported ADRs (TOP10).

Typical			Atypical			Total		
ADR	Frequency	Percentage	ADR	Frequency	Percentage	ADR	Frequency	Percentage
Extrapyramidal disorder	181	40.58%	Extrapyramidal disorder	766	21.84%	Extrapyramidal disorder	947	23.96%
Dystonia	29	6.50%	Akathisia	220	6.27%	Akathisia	235	5.94%
Tremor	26	5.83%	Constipation	194	5.53%	Constipation	198	5.01%
Akathisia	15	3.36%	White blood cell count decreased	147	4.19%	Drooling	156	3.95%
Abnormal sensation in eye	14	3.14%	Drooling	147	4.19%	White blood cell count decreased	152	3.85%
Drooling	9	2.02%	Tremor	98	2.79%	Tremor	124	3.14%
Hypertonia	9	2.02%	Hepatic function abnormal	78	2.22%	Somnolence	83	2.10%
Dry mouth	7	1.57%	Somnolence	77	2.20%	Hepatic function abnormal	80	2.02%
Somnolence	6	1.35%	Dizziness	74	2.11%	Dizziness	79	2.00%
Orthostatic hypotension	6	1.35%	Tachycardia	71	2.02%	Tachycardia	75	1.90%
Insomnia	6	1.35%	\	\	\	\	\	\

Except for 109 cases in which the outcomes were unknown, most patients (94.56%) improved or recovered after treatment or intervention while only one patient had sequelae. See Table 6 for details.

**TABLE 6:** Outcomes of the reported ADRs.

Variable	Frequency	Percentage (%)
Relieved	587	17.45
Cured	2,593	77.10
Not relieved	73	2.17
Left with sequelae	1	0.03
Missing	109	3.24

### Risk Factors of Serious ADRs

In this study, there were 298 (8.86%) reports of serious ADRs. The logistic regression analysis showed that the high-grade hospitals have a lower proportion of serious ADRs than low-grade hospitals (grade 2 adjusted OR 0.41, *p* = 0.002 and grade 3 adjusted OR 0.37, *p* = 0.002), and psychiatric hospitals reported a higher percentage of serious ADRs (adjusted OR 2.61, *p* < 0.001). Besides, compared with people aged 18 to 35, people younger than 18 years (adjusted OR 1.59, *p* = 0.008) and older than 64 years (adjusted OR 2.17, *p* = 0.011) were at higher risk of serious ADRs. Detailed results are shown in Table 7.

**TABLE 7:** Risk factors of serious ADRs.

Variable		P	Adjusted OR	95%CI
Female (refer to Male)		0.073	1.25	(0.980,1.589)
Age	18–35	0.002		
	18<	0.008	1.59	(1.128,2.247)
	35–65	0.869	0.98	(0.739,1.292)
	≥65	0.011	2.17	(1.189,3.942)
Season	spring	0.196		
	summer	0.788	0.95	(0.655,1.379)
	autumn	0.171	1.26	(0.905,1.748)
	winter	0.182	1.31	(0.882,1.937)
Atypical (refer to Typical)		0.056	1.55	(0.989,2.441)
Hospital level	1	0.006		
	2	0.002	0.41	(0.236,0.716)
	3	0.002	0.37	(0.198,0.705)
Psychiatric hospital (refer to General hospital)		<0.001	2.61	(1.684,4.043)
Multiple disease (refer to Single disease)		0.299	1.67	(0.634,4.410)
Polypharmacy (refer to Non-polypharmacy)		0.611	0.92	(0.677,1.258)

### Signal Mining Results

Through signal mining, the four methods obtained common 44 positive signals. Larger PRR values stand for the stronger association between the drug and ADR. Four off-label positive signals were found by comparing with the drug instructions. Sorted by PRR value, the top 10 positive signals and off-label positive signals were listed in Table 8. ROR (LI95), PRR (LI95), and IC(LI95) represent the lower limit of 95% confidence interval of ROR, PRR, and IC and superscript a represents off-label ADR.

**TABLE 8:** The signals of ADRs (4 methods, TOP10 and off-label).

Drug	ADR	ROR (LI95)	PRR	PRR (LI95)	χ2	IC(LI95)
perphenazine	Tongue induration	4.34	15.51	4.36	21.05	2.23
sulpiride	Insomnia	5.70	13.29	5.60	45.33	2.34
chlorpromazine	Blood pressure decreased	3.55	11.81	3.58	17.01	2.07
chlorpromazine	Orthostatic hypotension	4.79	10.63	4.70	37.87	2.17
chlorpromazine	Pruritus	3.38	11.19	3.41	16.04	2.01
haloperidol	Dystonia	6.66	9.54	6.14	136.25	2.05
olanzapine	Obesity	2.13	8.48	2.13	9.36	0.78
amisulpride	Pollakiuria^a^	2.00	7.95	2.00	7.74	1.03
chlorpromazine	Rash	2.74	7.46	2.76	14.91	1.62
haloperidol	Nuchal rigidity	2.57	7.32	2.57	14.50	1.21
ziprasidone	Dyspnoea^a^	1.64	5.07	1.65	7.27	0.77
quetiapine	Urinary incontinence^a^	1.41	5.28	1.41	4.62	0.65
olanzapine	Hepatic function abnormal^a^	2.17	3.18	2.12	33.54	0.80

The superscript a represents off-label adverse reactions.

## Discussion

Statistics showed that the number of ADR reports and the proportion of serious ADRs increased year by year from 2016 to 2020. One reason may be that the prevalence of mental disorders has been rising, leading to an increase in the use of APDs (Charlson et al., 2018; Huang et al., 2019). The other reason may be that the under-reporting and false-reporting problems in SRS had been improved, which is due to the application of CHPS and the improvement of the education level of medical staff (Lirong, 2021). In 2016, the National Center for ADR Monitoring established the Chinese Hospital Pharmacovigilance System (CHPS), which had been gradually promoted in China. By docking with hospital information systems and laboratory information systems, CHPS can detect ADR reports promptly, and realize the generation, review, report, feedback, and analysis of ADR information online, which improved the problem of under-reporting and false reporting and increase the number of reports. Under the influence of the Covid-19 epidemic, it is foreseeable that the incidence of mental disorders will further increase, and with the improvement of the adverse drug reaction monitoring system, the number of ADR reports of antipsychotic drugs will continue to increase, but the proportion of serious adverse reaction reports may decline.

In terms of the occurrence time, most ADRs occurred within 3 months after taking the medicine, which means patients and family members should take more responsibility for identifying ADRs. Close observation is required for the first 3 months after taking the medicine. The ADRs of antipsychotics mainly involved nervous system disorders, gastrointestinal disorders, and Cardiac disorders. The most common symptom of APDs was extrapyramidal disorder. Some scholars classified APDs as typical or atypical based on their liability to cause the extrapyramidal disorder (Meltzer, 2000). Compared with typical APDs, atypical APDs had a lower incidence of extrapyramidal side effects at conventional clinical doses (Meltzer, 2013). In this research, atypical APDs reported more reports and a higher proportion of serious ADRs. The reason may be that more patients use atypical APDs, and only patients in good condition use typical APDs. The symptoms caused by typical APDs and atypical APDs were different. Dyskinesia was more common in typical APDs, such as dystonia and hypertonia. Some research shows that the incidence of tardive dyskinesia was twenty percent in patients using typical APDs, and only one or two percent in patients using atypical APDs (Al Hattab et al., 2018). For patients with typical APDs, tardive dyskinesia should be given special attention, because it is not easy to detect, usually insensitive to treatment, and may be permanent.

Most patients were improved or cured after treatment and intervention while only one patient had sequelae. This patient was diagnosed with schizophrenia and developed sinus bradycardia after taking sulpiride tablets. The doctor believed that continuing to take this drug would benefit the patient more than harm, so this patient continued to use it until leading to long-term sinus bradycardia. We suggested that it might be wiser to replace the drug after a serious ADR occurred.

Our research showed that low-level hospitals, psychiatric hospitals, youth, and old age were risk factors for serious ADRs. Many studies had shown that adolescents have a higher risk of ADRs while using APDs (Safer, 2004; Correll, 2008; Sikich et al., 2008). Sikich found that weight gain and extrapyramidal effects were more common and severe in adolescents treated with risperidone and olanzapine (Sikich et al., 2004). The elderly should be extra careful when using APDs as well. Research performed by Daniel showed that older age is a risk factor for death from the neuroleptic malignant syndrome, which is a potentially fatal idiosyncratic reaction caused by APDs (Guinart et al., 2021). There may be two reasons for the lower proportion of severe ADR in high-level hospitals. On the one hand, high-level hospitals have more experienced doctors and nurses who will pay close attention to patients so they could find ADR early and avoid the occurrence of severe ADR. On the other hand, the strict ADR monitoring and reporting system makes high-level hospitals rarely underreport. On the contrary, lack of staff and imperfect systems in low-level hospitals are the main reasons for the high proportion of serious ADRs. Psychiatric hospitals have reported a higher proportion of severe ADRs, which may be due to the fact that they have more severe psychiatric patients. In China, the general hospital psychiatric units and psychiatric hospitals are the main provider of mental health services, and psychiatric hospitals usually provide intensive services for severe psychiatric disorders (Chen et al., 2013). As patients with severe illness often require high-dose medications, they are more likely to develop severe ADR.

44 Drug-ADR combinations were identified as positive signals by all four signal mining methods. A positive signal indicates that this combination is significantly higher and reaches the threshold compared to the background frequency. If the signal included an off-label ADR, it is necessary to do further analysis to prevent potential drug safety incidence. By comparing with drug instructions, we found four off-label signals, including amisulpride-pollakiuria, ziprasidone-dyspnoea, quetiapine-urinary incontinence, olanzapine-hepatic function abnormal. We have not found any reports of amisulpride-related pollakiuria in medical literature, but Mendhekar and Lohia (2009) and Niranjan et al. (2017) respectively reported a case of amisulpride causing urinary incontinence (Mendhekar and Lohia, 2009; Niranjan et al., 2017). Considering that pollakiuria could cause urge urinary incontinence, we suspected that pollakiuria might be an early symptom of urinary incontinence caused by amisulpride. Further research on amisulpride-related pollakiuria or urinary incontinence requests more case reports. Tsai and Harada separately reported one case of dyspnoea after ziprasidone administration (Tsai et al., 2008; Harada et al., 2018). This study found four cases of dyspnoea caused by ziprasidone and considered ziprasidone-dyspnoea as a positive signal. The mechanism of ziprasidone inducing dyspnea is to cause respiratory muscle or laryngeal dystonia, and timely withdrawal and treatment with anticholinergic agents can help alleviate the symptoms (Tsai et al., 2008). Elyasi reported two cases of urinary incontinence caused by quetiapine in patients with bipolar disorder (Elyasi and Darzi, 2017). Between July 2011 to July 2018, The National Coordination Centre-Pharmacovigilance Programme of India (NCC-PvPI)received six reports of quetiapine-induced urinary incontinence. Experts at the NCC-PVPI analyzed six reports and found that there was a strong causal relationship between quetiapine and urinary incontinence. Thus they recommended that the instructions of quetiapine should be modified to treat urinary incontinence as a clinically significant ADR (World Health Organization, 2019). Urinary incontinence is a serious and embarrassing side effect, which adversely affects the patients’ quality of life and compliance. Therefore, we think the doctors and patients should be warned about the risk of quetiapine-related urinary incontinence. There were many reports of olanzapine-related hepatic function abnormality (Farooque, 2003; Kahn, 2014; Katagiri et al., 2018). It is generally believed that olanzapine could cause an isolated asymptomatic increase in the aminotransferase levels, but Lam reported a case of a 17-year-old man with first-episode schizophrenia who developed olanzapine-induced hepatitis, cholestasis, and splenomegaly, indicating that olanzapine could cause much liver damage (Lui et al., 2009). The mechanism of olanzapine-induced liver dysfunction remains unclear. A study by TingJiang showed that up-regulation of FATP2/FABP1 and down-regulation of hepatic OCTN2 probably contribute to olanzapine-induced liver steatosis (Jiang et al., 2019).

In general, we performed a safety evaluation of antipsychotic drugs by analyzing a database of the provincial spontaneous reporting system. As clinical trials were carried out under specific conditions and usually couldn’t include adequate people, our research plays an important role in evaluating the safety of antipsychotic drugs. The statistical results and ADR signals obtained in this study are helpful in guiding the safe use of antipsychotics, and might be clues for ADR mechanism research, even providing advice for modifying drug labels based on the detection of off-label ADRs.

This study has potential limitations. Firstly, the results were biased due to the inevitable under-reporting and false-reporting in SRS. Secondly, the signal detection method is based on the reported quantitative correlation rather than biological correlation, and cannot represent the inevitable causal relationship between drugs and adverse reactions. However, based on the following considerations, our research still was important. The development of CHPS had greatly reduced the under-reporting and false-reporting, so the bias caused by data has been reduced as much as possible. Although we couldn’t prove the causal relationship between drugs and ADRs, the positive signals could provide clues for further research. At the same time, we provide relevant literature to support the off-label signals. Overall, this study has a positive impact on promoting the rational use of APDs.

## Conclusion

In this research, we found that most ADRs occurred within 3 months after taking the medicine, so close observation was required for the first 3 months. The ADRs of antipsychotics involved multiple organ-system damages but were not severe, most patients were improved or cured after treatment and intervention while only one patient had sequelae. But it might be wiser to replace the drug after a serious ADR occurred. The symptoms caused by typical APDs and atypical APDs were different. For patients with typical APDs, dyskinesia was more common and should be given special attention. Statistics showed that low-level hospitals, psychiatric hospitals, youth, and old age were risk factors for serious ADRs. The four off-label signals obtained by signal mining should be paid special attention, including amisulpride-pollakiuria, ziprasidone-dyspnoea, quetiapine-urinary incontinence, and olanzapine-hepatic function abnormal.

## Data Availability

The data analyzed in this study is subject to the following licenses/restrictions: Data may be obtained from a third party and are not publicly available. Requests to access these datasets should be directed to fengda@hust.edu.cn.
